# Chemical Exchange Saturation Transfer (CEST) Signal at −1.6 ppm and Its Application for Imaging a C6 Glioma Model

**DOI:** 10.3390/biomedicines10061220

**Published:** 2022-05-24

**Authors:** Qi-Xuan Wu, Hong-Qing Liu, Yi-Jiun Wang, Tsai-Chen Chen, Zi-Ying Wei, Jung-Hsuan Chang, Ting-Hao Chen, Jaya Seema, Eugene C. Lin

**Affiliations:** 1 Department of Chemistry and Biochemistry, National Chung Cheng University, Chiayi 62102, Taiwan; qaz950270@gmail.com (Q.-X.W.); henry84515@gmail.com (H.-Q.L.); q60501@gmail.com (Y.-J.W.); zzzei1112@gmail.com (Z.-Y.W.); ruby19960814@gmail.com (J.-H.C.); rroee135@gmail.com (T.-H.C.); 2 Institute of Biomedical Sciences, Academia Sinica, Taipei 11529, Taiwan; hazelnut.chen.scu@gmail.com (T.-C.C.); dmjseema2k@gmail.com (J.S.); 3 Center for Nano Bio-Detection, National Chung Cheng University, Chiayi 62102, Taiwan

**Keywords:** magnetic resonance imaging (MRI), chemical exchange saturation transfer (CEST), relayed nuclear Overhauser effect (rNOE), cholesterol, glioma

## Abstract

The chemical exchange saturation transfer (CEST) signal at −1.6 ppm is attributed to the choline methyl on phosphatidylcholines and results from the relayed nuclear Overhauser effect (rNOE), that is, rNOE(−1.6). The formation of rNOE(−1.6) involving the cholesterol hydroxyl is shown in liposome models. We aimed to confirm the correlation between cholesterol content and rNOE(−1.6) in cell cultures, tissues, and animals. C57BL/6 mice (N = 9) bearing the C6 glioma tumor were imaged in a 7 T MRI scanner, and their rNOE(−1.6) images were cross-validated through cholesterol staining with filipin. Cholesterol quantification was obtained using an 18.8-T NMR spectrometer from the lipid extracts of the brain tissues from another group of mice (N = 3). The cholesterol content in the cultured cells was manipulated using methyl-β-cyclodextrin and a complex of cholesterol and methyl-β-cyclodextrin. The rNOE(−1.6) of the cell homogenates and their cholesterol levels were measured using a 9.4-T NMR spectrometer. The rNOE(−1.6) signal is hypointense in the C6 tumors of mice, which matches the filipin staining results, suggesting that their tumor region is cholesterol deficient. The tissue extracts also indicate less cholesterol and phosphatidylcholine contents in tumors than in normal brain tissues. The amplitude of rNOE(−1.6) is positively correlated with the cholesterol concentration in the cholesterol-manipulated cell cultures. Our results indicate that the cholesterol dependence of rNOE(−1.6) occurs in cell cultures and solid tumors of C6 glioma. Furthermore, when the concentration of phosphatidylcholine is carefully considered, rNOE(−1.6) can be developed as a cholesterol-weighted imaging technique.

## 1. Introduction

Metabolite-weighted magnetic resonance imaging (MRI) can be performed based on chemical exchange saturation transfer (CEST), which allows us to monitor muscle [1], the liver [2], ischemic stroke [3], and cancers [4,5,6]. A CEST effect relies on the radiofrequency (RF) pulse to saturate protons at varying frequency offsets, which usually range between −5 and 5 ppm relative to water resonance. When the exchangeable protons (e.g., amide, amine, and hydroxyl) on metabolites are saturated, the magnitude of a water signal can be reduced through the exchange of protons between water molecules and metabolites. With this saturation and exchange mechanism, metabolites and other biomolecules (in the millimolar concentration range) can be indirectly detected by reducing the water signal [7,8]. 

The effects of CEST can be plotted on a z-spectrum, in which the x-axis indicates the saturation offsets that are based on a water resonance of 0 ppm, and the y-axis indicates the magnitude of saturation at an offset as obtained by normalizing the intensity of water resonance to an unsaturated level [7,8]. However, a z-spectrum is highly subject to interference from direct water saturation and magnetization transfer (MT), which are usually more substantial than CEST dips. Researchers have proposed several methods for removing the background signals in CEST through pulse sequences (e.g., chemical exchange rotational transfer (CERT) [9,10,11] and variable delay multi-pulse (VDMP) [12,13]) or numerical methods (asymmetric MT [1,6,7,14,15], extrapolated semisolid magnetization transfer reference (EMR) [15,16], and deconvolutions with one or multiple Lorentzian functions [2,17,18,19,20,21,22]). These procedures are crucial for small CEST signals [17], such as the relayed nuclear Overhauser effect at −1.6 ppm, that is, rNOE(−1.6) [9,17,19,20,21,23,24,25,26]. 

The nuclear Overhauser effect (NOE) is caused by the cross-relaxation of nuclear spins, which results from the interactions between nuclear dipoles. NOEs can only be observed when the distance between two protons is sufficiently close (approximately < 5 Å) [27], and connections through chemical bonds are not required. When an unexchangeable proton is saturated, its neighboring protons can also be saturated through an NOE network. Because exchangeable protons are saturated through NOEs, bulk water can be saturated via the proton exchange last. This entire process is called relayed NOE (rNOE) [28]. The most prominent rNOE occurs at −3.5 ppm, and it is attributed to aliphatic protons. Although the rNOE at −3.5 ppm has less chemical specificity, it can be utilized to identify brain tumors [18,22,23,29]. By contrast, other rNOEs have a higher specificity, including the down-field rNOE at 3.5 ppm that is attributed to aromatic protons [30] and the rNOE at −1 ppm that is attributed to glycoNOE [2]. The rNOE(−1.6) was first revealed to be diminished in a 9L rat brain tumor model [9] and an ischemic stroke rat model [24]. The chemical origin of rNOE(−1.6) is presumably the choline methyl protons on phosphatidylcholines. The choline methyl group lacks exchangeable protons, and therefore the rNOE pathway can only be completed through the hydroxyls from nearby cholesterols. This dependence on cholesterols to form rNOE(−1.6) was observed in liposome models [25,26]. 

Approximately 20–25% of lipids in cell membranes are cholesterols [31], and therefore, the demand for cholesterol is high during the proliferation and progression of tumor cells [32,33,34]. Cholesterols can assemble with sphingomyelins to form lipid rafts on cell membranes such that the activity levels of membrane proteins could be regulated. For instance, the serine/threonine protein kinase, Akt, which is crucial to apoptosis and abnormal growth, is activated through an association with the lipid rafts [35,36]. Numerous hormones or oxysterols that are essential to promoting tumor growth (e.g., estrogen, progestogen, 24-hydroxylchoeslterol, and 27-hydroxylchoeslterol) utilize cholesterols as precursors [32,33]. The cholesterol homeostasis that occurs in the brain is a unique process. The blood–brain barrier blocks the cholesterol synthesized from the liver, and hence the synthesis of cholesterol primarily relies on the astrocytes in the brain [37,38]. Excess cholesterol in the brain is delivered to the peripheral system through high-density lipoproteins. Glioma cells have been demonstrated to detour around de novo cholesterol synthesis and to obtain cholesterols by upregulating low-density lipoprotein (LDL) receptors to uptake cholesterol from nearby astrocytes [38]. 

Numerous studies have suggested that rNOE(−3.5) imaging can be used to identify tumor regions [18,22,23,29]; however, rNOE(−3.5) may have a limited chemical/metabolic specificity. By contrast, rNOE(−1.6) originates from the choline methyls on phosphatidylcholines and requires the hydroxyl in cholesterol [26]. This unique mechanism can reveal the alternation that occurs within biological membranes and provide specific insights at the molecular level. The present study integrated the rNOE(−1.6) images obtained from mice, the cholesterol staining of brain sections, and the rNOE(−1.6) signals from cell homogenates. Additionally, the cholesterol content of the lipid extracts of tissues and cell homogenates was quantified through ^1^H nuclear magnetic resonance (NMR). On the basis of the results for the C6 cell cultures and C6 gliomas obtained from mice brains, the experiments of the present study were conducted to establish a foundation for cholesterol-weighted imaging.

## 2. Method

### 2.1. Cell Cultures, Homogenates, and Lipid Extraction

C6 cells were cultured using Dulbecco’s Modified Eagle’s Medium (DMEM, D7777, Sigma-Aldrich, Saint Louis, MO, USA) buffer with an additional 10% fetal bovine serum (TMS-013-BKR, Merck, Darmstadt, Germany) and 1% penicillin-streptomycin (P0781, Sigma-Aldrich, Saint Louis, MO, USA) at 37 °C with 5% CO_2_. The cell cultures generally required three to four days to reach 70% confluence. Approximately 8 × 10^7^ cells were required to prepare a phantom of cell homogenates. Two solutions were prepared to manipulate the cholesterol content in the cell cultures [39,40], namely (1) a methyl-β-cyclodextrin (MβCD, 332615, Sigma-Aldrich, Saint Louis, MO, USA) solution (500 mM in phosphate-buffered saline [PBS] buffer) for cholesterol-depletion experiments and (2) a chol-MβCD solution comprising 85% (*v*/*v*) MβCD solution (240 mM in PBS) and 15% (*v*/*v*) cholesterol solution (25 mg/mL in ethanol) for cholesterol-enrichment experiments. 

Each cultured medium was removed and washed with PBS twice before cholesterol manipulation. A total of 10 mL of fresh DMEM was added together with an additional 200 μL of MβCD solution and 250 μL of chol-MβCD solution for cholesterol depletion and enrichment, respectively; the control group was treated with 10 mL of fresh DMEM. Each group was cultured at 37 °C for 30 min and subsequently detached with trypsin (15090-046, ThermoFisher, Waltham, MA, USA). The harvested cells were washed with PBS twice and collected through centrifugation. Each cell pellet was resuspended in 1 mL of deionized water and frozen in liquid nitrogen to lyse the cells. Cell debris was homogenized in an ice bath by using a sonicator (20 s on and 20 s off for 3 cycles). The homogenates were lyophilized and subsequently resuspended in 405 μL of PBS and 45 μL of D_2_O (151882, Sigma-Aldrich, Saint Louis, MO, USA) to allow for T_1_ and CEST measurements.

Cholesterol concentration was estimated by examining the lipid extracts of the cell homogenates [41]. After the T_1_ and CEST experiments were completed, the cell homogenates were removed from the NMR tubes that they were housed in and lyophilized. In total, 200 μL of methanol and 85 μL of deionized water were added to the dried homogenates, and the resulting solution was sonicated (20 s on and 20 s off for 5 cycles). Next, 200 μL of chloroform was added to the methanol–water solution, which was then vortexed for 2 min. Finally, 200 μL of chloroform and 200 μL of water were added to the resulting solution, which was vortexed again for 2 min. The organic/aqueous mixture was cooled in an ice bath for 10 min and centrifuged for 15 min at 12,000× *g* and 4 °C. The organic layer was carefully transferred out, and the organic solvent was removed using N_2_ streams and lyophilization. The sample was redissolved in 450 μL of D-chloroform (612200, Sigma-Aldrich, Saint Louis, MO, USA) to prepare them for ^1^H NMR measurements. 

### 2.2. Animals, Section Staining, and Tissue Extraction

The procedures for performing animal experiments were approved by the Institute of Animal Care and Utilization Committee of Academia Sinica, Taipei, Taiwan (protocol code number: 17-02-1050 and the approved date: 25 Auguest 2021). C57BL/6 male mice (6 weeks old) were purchased from BioLASCO (Taiwan) and raised with free access to water and food on a 12-h day/12-h night cycle. In total, 5 × 10^5^ C6 cells suspended in 2 μL of PBS buffer were injected into the right hemisphere at a flow rate of 500 nL/min to establish a tumor model. The mice were imaged three days after the aforementioned surgery. 

The mice were anesthetized with 0.16 mL of a solution comprising Zoletil 50 (sagarpa-Q-0042-058, Virbac, Carros, France), Xylazine (08443, Elite Bio-Science, New Orleans, LA, USA), and saline at a volume ratio of 1:2:12 to prepare the brain section. The mice were perfused with saline until the resulting effluent was clear, and they were then perfused with 20 mL of 4% paraformaldehyde (PFA, 158127, Sigma-Aldrich, Saint Louis, MO, USA). Each mouse brain was removed and immersed in 4% PFA at 4 °C for one day, after which it was transferred to a 20% sucrose solution followed by a 30% sucrose solution to prevent the formation of ice crystals that can damage tissue morphology. Finally, 20-μm tissues were obtained through frozen sectioning. The optimal cutting temperature compound (4583, Sakura, Torrance, CA, USA) that was used during the sectioning procedure was removed by using PBS before staining. Each tissue section was immersed in a hematoxylin buffer (HHS16, Sigma-Aldrich, Saint Louis, MO, USA) for 5 min, differentiated with acid alcohol, and washed with water. A four-percent PFA solution was used to fix the section, and the excess PFA was removed by immersing the section in PBS for 5 min and in glycine (G7403, Sigma-Aldrich, Saint Louis, MO, USA) for another 5 min. Each section was covered with a 0.1-mL filipin (F4767, Sigma-Aldrich, Saint Louis, MO, USA) solution for 2 h in a dark environment for cholesterol staining [42,43]. Excess filipin solution was removed by immersing each section in fresh PBS buffer for 3 min twice. Each section was then sealed with CC/mount medium (C9368, Sigma-Aldrich, Saint Louis, MO, USA).

Mouse brains were removed immediately following scarification on days nine and 12, and the tumor and normal parts of each brain were separated visually. These tissues were prepared for the lipid extraction of tissues. The wet weights of tumor and normal tissues ranged between 3.2 and 28.7 mg and between 26.1 and 42.9 mg, respectively. These tissues were subsequently vortexed in an iced solution (4 mL of methanol per gram of tissue and 0.85 mL of water per gram of tissue) until the solution became homogenous. The solution was then vortexed with an additional 2 mL of chloroform per gram of tissue. Finally, 2 mL of chloroform per gram of tissue and 2 mL of water per gram of tissue were added to the solution, which was then vortexed. The organic/aqueous mixture was cooled down in an ice bath for 10 min and then centrifuged for 15 min at 12,000× *g* and 4 °C. The organic layer was carefully transferred out, and the organic solvent was removed through N_2_ streams and lyophilization. The dried extracts, which were each equivalent to 1.2 mg of wet tissue in weight, were redissolved in 550 μL of D-chloroform (DLM-7TB, Cambridge Isotope Laboratories, Tewksbury, MA, USA) for ^1^H NMR experimentation. 

### 2.3. MRI

The images were obtained using a 7-T Bruker BioSpec 70/20 USR system equipped with an RF transmit coil (RF RES 300 1H 112/086 QSN TO AD) and a receiver coil (RF ARR 300 1H M.BR. 2 × 2 RO AD). For all images, a readout sequence of rapid imaging with refocused echoes (RAREs) was utilized, with the field of view being 16 × 16 mm^2^, slice thickness being 2 mm, and average being 1. A T_1_ map was generated using the sequence “RARE VTR” with a RARE factor of 3; TE of 14 ms; TR values of 50, 200, 500, 800, 1000, 2000, 4000, and 6000 ms; and a matrix size of 128 × 64. CEST and water saturation shift referencing (WASSR) [44] images were acquired with a dummy scan of 4, RARE factor of 16, TE/TR of 42.07/5000 ms, and matrix size of 128 × 64. For the WASSR experiments [44], the saturation duration and a saturation amplitude were 4 s and 0.1 μT, respectively. The saturation offsets were between −1 and 1 ppm (with a 0.1-ppm increment). The saturation duration was 4 s, and saturation amplitudes were 0.3, 0.6, and 0.9 μT. The saturation offsets were 333, −10, −8, −6, −5, −4 to 4 (with a 0.1-ppm increment), 5, 6, 8, and 10 ppm.

### 2.4. NMR

The z-spectra of cell homogenates were measured using a Bruker 400-MHz (9.4 T) AVIII spectrometer and a BBO 400-MHz S1 5-mm probe with a z-gradient. The T_1_ values of the homogenates were measured using the inversion-recovery sequence t1ir, and the following parameters were applied: spectral width = 7.5 ppm, number of sampling points = 16,384, acquisition time = 2.7 s, recycle delay = 5 s, dummy scan = 4, and signal average = 4. The durations of inversion recovery were 0.01, 0.05, 0.1, 0.25, 0.5, 1, 2, 4, 6, and 10 s. The CEST results pertaining to the cell homogenates were obtained using the sequence stddiff, and the following acquisition parameters were applied: spectral width = 10 ppm, number of sampling points = 11,998, acquisition time = 1.5 s, recycle delay = 2 s, dummy scan = 4, signal average = 1. The saturation amplitudes were 0.3, 0.6, and 0.9 μT, and the saturation duration was 5 s. For the CEST experiments, the saturation offsets were implemented as follows: 250, −20, −15, −10, −6, −5, −4.5, −4 to −3 (with a 0.1-ppm increment), −2.8 to −2 (with a 0.2-ppm increment), −1.9 to −1 (with a 0.1-ppm increment), −0.8 to 4 (with a 0.2-ppm increment), 4.5, 5, 6, 10, 15, and 20 ppm. The ^1^H spectra of the homogenate extracts were acquired using the sequence zg30, and the following parameters were applied: spectral width = 20 ppm, number of sampling points = 32,768, acquisition time = 2 s, recycle delay = 2 s, and signal average = 64. 

The ^1^H spectra of the tissue extracts were obtained using an Avance 800 MHz spectrometer and a TX1 5-mm CryoProbe probe with a z-gradient by using the following parameters: spectral width = 14 ppm, number of sampling points = 32,768, acquisition time = 1.46 s, recycle delay = 2 s, and signal average = 512.

### 2.5. Data Processing

#### 2.5.1. MRI

The *T*_1_ value of each voxel was determined using the saturation recovery function as follows:

(1) $$I\left(t\right)=I_{0}\left(1-\exp\left(-t/T_{1}\right)\right),$$

where *I*(*t*) is the measured intensity of a pixel with a recovery delay of *t*, and *I*_0_ is the intensity of a pixel with no recovery delay. B_0_ correction was performed using the WASSR method [44], in which a Lorentzian function was fitted to the saturation spectrum, and the B_0_ offset was determined by obtaining the center of the fitted Lorentzian function. The CEST images were normalized to the image measured at 333 ppm and subsequently smoothed using a 3 × 3 median filter. A total of 25 voxels from normal regions and another 25 voxels from tumor regions were selected and averaged to obtain the representative z-spectra of each animal. The interferences from water, MT, and *T*_1_ were minimized by employing the residuals of apparent exchange-dependent relaxation rate (*AREX_resid_*) [17,18,19,20,21,22]. In brief, *AREX_resid_* was obtained using the inversed residue between the measured z-spectrum (*S_meas_*) and fitted z-spectrum (*S_fit_*), which was corrected by *T*_1_ and the pool size of MT (*f_m_*); the equation for obtaining *AREX_resid_* is as follows:

(2) $$AREX_{resid}\left(\Delta \omega \right)=\left(\frac{S_{0}}{S_{meas}\left(\Delta \omega \right)}-\frac{S_{0}}{S_{fit}\left(\Delta \omega \right)}\right)\times \frac{1}{T_{1}}\left(1+f_{m}\right).$$

where *S*_0_ is the normalizer, and ∆*ω* is the saturation offset. The obtained *AREX_resid_*(−3.5) was further isolated using a Gaussian function centered at approximately −3.5 ppm; the area under the curve (AUC) of the Gaussian function was referred to as the size of *AREX_resid_*(−3.5). The residual signals from Gaussian deconvolution were summed, determined to be between −1.3 and −1.9 ppm, and used as the AUC of *AREX_resid_*(−1.6). The fitting procedures that were performed for *AREX_resid_* and *AREX_resid_*(−3.5) isolation are described in detail in the Supplementary Materials.

#### 2.5.2. NMR

The inversion recovery curve for the cell homogenates indicated the presence of multiple *T*_1_ relaxations. Therefore, we fitted the normalized recovery curve by applying the following equation (which includes two *T*_1_*s*):

(3) $$I\left(t\right)=1-2\left(x_{a}\exp\left(-\frac{t}{T_{1,a}}\right)+\left(1-x_{a}\right)\exp\left(-\frac{t}{T_{1,b}}\right)\right)$$

where *T*_1,a_ and *T*_1,b_ are the *T*_1_ relaxations for pools *a* and *b*, respectively, and *x_a_* is the portion of the pool *a*. The averaged *T*_1_ in Equation (4) is the *T*_1_ for a cell homogenate. Equation (4) is expressed as follows:

(4) $$T_{1,avg}=x_{a}T_{1,a}+\left(1-x_{a}\right)T_{1,b}$$

The z-spectra were obtained by normalizing the water resonance to the acquisition at 250 ppm. The *AREX_resid_* of the cell homogenates was obtained using Equation (2), and *AREX_resid_*(−1.6) and *AREX_resid_*(−3.5) were isolated using two Gaussian functions. The areas of these two Gaussian functions served as the AUCs of *AREX_resid_*(−1.6) and *AREX_resid_*(−3.5). The procedures that were performed for AREX correction and rNOE isolation (i.e., isolation from the AREX residual) are described in detail in the Supplementary Materials.

#### 2.5.3. Cholesterol Quantification through ^1^H NMR

The D-chloroform solvent contained internal standard tetramethylsilane (TMS) at a concentration of 0.05% *v*/*v* (3.67 mM). The AUC of ^1^H NMR resonance was assumed to be proportional to its concentration. Therefore, cholesterol concentration was estimated by comparing the AUC of cholesterol methyl (0.68 ppm) to that of TMS at 0 ppm.

## 3. Results

**Figure 1:**
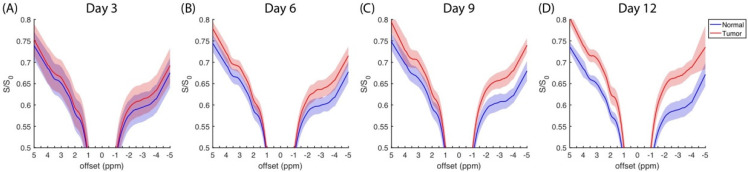
Average z-spectra (N = 9) from normal (blue) and tumorous (red) brain as measured on days 3 (**A**), 6 (**B**), 9 (**C**), and 12 (**D**). Shading areas indicate standard deviation resulting from animal variation. Saturation amplitude and saturation duration are 0.9 μT and 4 s, respectively.

The average z-spectra (N = 9) that was measured at four days at a saturation amplitude of 0.9 μT are plotted in Figure 1, in which the normal and tumor sides are represented by the blue and red regions, respectively. The z-spectra obtained at saturation amplitudes of 0.3 and 0.6 μT are plotted in Figure S1. Typical features were identified in the distinct amide (3.5 ppm), amine (2 ppm), and rNOE (−3.5 ppm) dips and the MT background. A minor absorption appeared on the shoulder of the direct saturation at −1.6 ppm, and it was referred to as the choline group from lipids. The normal side had a higher saturation than the tumor side [9,19,25], and this difference increased during the experimental period.

**Figure 2:**
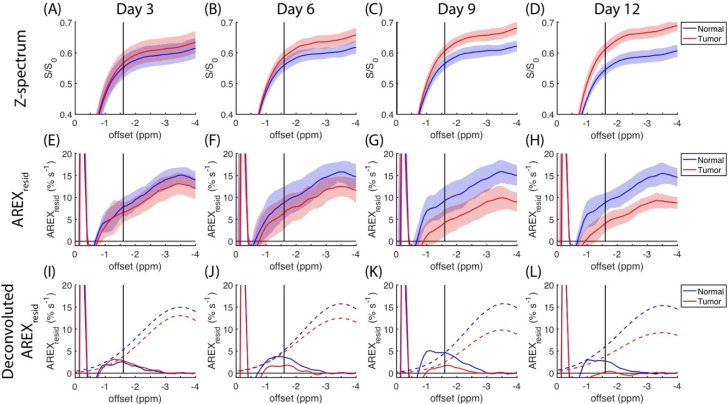
Expansion of z-spectra from Figure 1 for comparative purposes (**A**–**D**); the corresponding residuals of apparent exchange-dependent relaxation rate (*AREX_resid_*) are plotted from (**E**–**H**), which include averaged signals (N = 9) from normal (red) and tumorous (blue) brain tissues and their standard deviations (shaded areas). Averaged *AREX_resid_* signals from (**E**–**H**) are deconvoluted using a Gaussian function centered at approximately −3.5 ppm (**I**–**L**), in which dashed lines indicate rNOE(−3.5) and solid lines indicate residues from deconvolutions.

**Figure 3:**
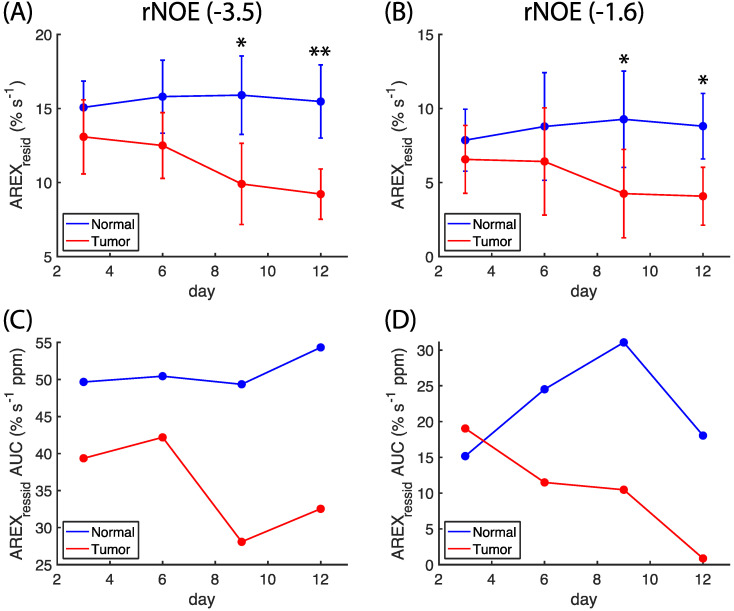
Longitudinal changes in magnitudes of *AREX_resid_* at −3.5 ppm (**A**) and −1.6 ppm (**B**), as extracted from Figure 2 (N = 9); longitudinal changes in area under the curve (AUC) of corresponding *AREX_resid_* at −3.5 ppm (**C**) and −1.6 ppm (**D**). (* *p* < 0.05, ** *p* < 0.001).

The region of negative offset in the z-spectra is expanded in Figure 2A–D, and the same region of *AREX_resid_* is plotted in Figure 2E–H. To further elucidate the changes to *AREX_resid_*(−1.6), a Gaussian function was fitted to the average *AREX_resid_* centered at approximately −3.5 ppm. In Figure 2I–L, *AREX_resid_*(−3.5) is indicated by dashed lines, and the residuals are indicated by solid lines. These residuals contain a signal that was approximately between −2 and −1 ppm. The information on *AREX_resid_*(−3.5) and *AREX_resid_*(−1.6) that is plotted in Figure 2 is further organized in Figure 3A,B. The amplitudes of *AREX_resid_*(−3.5) and *AREX_resid_*(−1.6) on the normal side remained mostly unchanged between days three and 12; however, the amplitudes of *AREX_resid_*(−3.5) and *AREX_resid_*(−1.6) on the tumor side were lower on days 9 and 12. The *AREX_resid_* values at −1.6 and −3.5 ppm indicated a significant difference (*p* < 0.05 or 0.001) between the normal and tumor tissues on days nine and 12. The AUCs of *AREX_resid_*(−3.5) and *AREX_resid_*(−1.6) were greater on the normal side than on the tumor side, and the AUC of *AREX_resid_*(−1.6) tended to decrease during the growth of the tumor (Figure 3C,D). However, these AUCs were obtained from averaged *AREX_resid_* values, and we could not analyze the difference between the tissues and the longitudinal changes to the statistical metrics.

**Figure 4:**
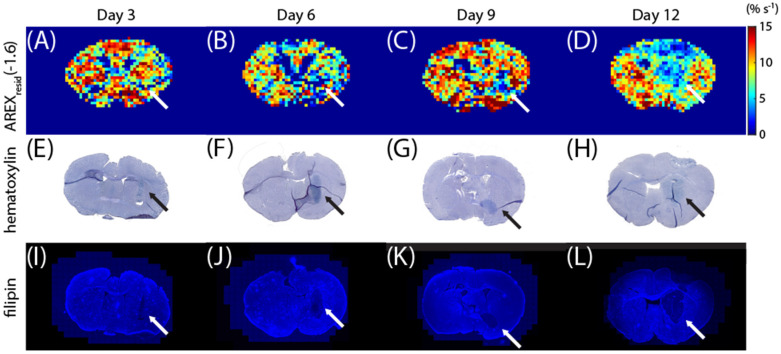
Images of *AREX_resid_*(−1.6) (**A**–**D**), hematoxylin staining (**E**–**H**), and filipin staining (**I**–**L**) on days 3, 6, 9, and 12. The arrows indicate the tumor regions.

Additional mice were sacrificed on days 3, 6, 9, and 12 to correlate the *AREX_resid_*(−1.6) images with the staining images. The *AREX_resid_*(−1.6) images are shown in Figure 4A–D, and they indicate that rNOE(−1.6) was hypointense in the tumor region, particularly on days 9 and 12. The brain sections were stained using hematoxylin (Figure 4E–G) and filipin (Figure 4I–L). For hematoxylin staining, the dense region of nuclei (which indicate tumors [45,46]) approximately coincides with the tumor region from the rNOE(−1.6) images. The filipin staining results further suggest that the C6 tumor region was cholesterol deficit [42]. The *T*_2_-weighted, *T*_1_ map, MT, and *AREX_resid_* (at 3.5, 2, −1.6, and −3.5 ppm) images obtained from these mice are shown in Figure S2.

**Figure 5:**
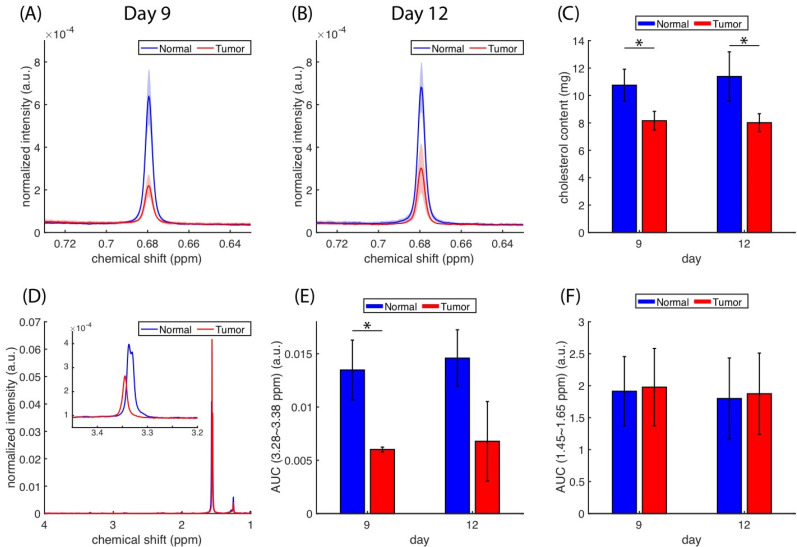
^1^H NMR of cholesterol methyl (0.68 ppm) from lipid extracts of normal and tumorous brain tissues (N = 3) on days nine (**A**) and 12 (**B**) and their cholesterol content (**C**) as quantified using AUCs of methyl shown in (**A**,**B**). (**D**) The choline methyls from the phosphatidylcholines (~3.35 ppm) and alkyl hydrogens (~1.5 ppm) can be identified in the ^1^H spectra. The AUCs between 3.28 to 3.38 ppm and 1.45 to 1.55 ppm were used to estimate the relative amounts of phosphatidylcholine (**E**) and total lipid (**F**). (* *p* < 0.05).

Cholesterol quantification was performed using another set of mice (N = 3), specifically the ^1^H NMR spectra of their brain extracts. Compared with standard cholesterol spectra (Figure S3A,B), the peaks at 1.00 and 0.68 ppm can be attributed to the presence of sterol methyls in the cholesterol. The resonance at 0.68 ppm (Figure 5A,B) was lower in tumors than in normal tissues. The whole spectra of the lipid extracts are presented in Figure S3C–F. We utilized the AUCs of cholesterol methyl (at 0.68 ppm) to estimate the cholesterol content in the tissue data plotted in Figure 5C. A significant difference (*p* < 0.05) in cholesterol content was identified between normal and tumor tissues, but no significant change was detected longitudinally. The ^1^H spectra (Figure 5D) from the tumor and normal extracts could also reveal the lipid composition, in which the peaks located around 3.35 and 1.5 ppm can be attributed to choline methyls from the phosphatidylcholines and alkyl hydrogens [47]. Figure 5E shows that the relative contents of phosphatidylcholines were lower in tumors than in normal tissues based on the integration from 3.28 to 3.38 ppm. The amounts of total lipid estimated based on the alkyl hydrogens (from 1.45 to 1.65 ppm) were roughly the same in tumors and normal tissues (Figure 5F).

**Figure 6:**
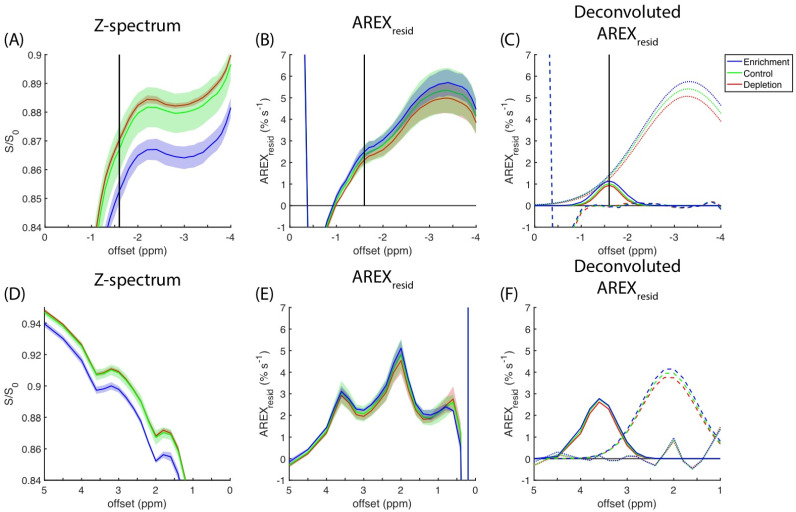
Averaged (N = 3) z-spectra (**A**,**D**) from cholesterol-depleted (red), control (green), and cholesterol-enriched (blue) cell homogenates, corresponding *AREX_resid_* (**B**,**E**), and deconvoluted *AREX_resid_* (**C**,**F**). Standard deviations are indicated by shaded areas. Dotted lines indicate rNOE(−3.5), solid lines indicate rNOE(−1.6), and dashed lines indicate residues from deconvolutions. The CEST signals from the negative and positive regions are shown in (**A**–**C**) and (**D**–**F**), respectively.

The CEST experimental results for the cell homogenates measured at a 0.9-μT saturation amplitude are presented in Figure 6A. The magnitude of the rNOE dips in the z-spectra increases with cholesterol content (i.e., cholesterol enrichment > control > cholesterol depletion). The full z-spectra of the cell homogenates obtained at amplitudes of 0.3 and 0.6 μT are presented in Figure S4. Figure 6B reveals a positive correlation between the amplitude of rNOE(−1.6) and cholesterol content remains on the basis of the *AREX_resid_* metric. The deconvoluted *AREX_resid_* that used two Gaussian functions indicated that *AREX_resid_*(−3.5) and *AREX_resid_*(−1.6) were both dependent on the level of cholesterol content (Figure 6C). The z-spectra and *AREX_resid_* results pertaining to the amides and amines that responded to cholesterol manipulation are presented in Figure 6D,E, respectively. The deconvolutions of the amide and amine pools in *AREX_resid_* are presented in Figure 6F. 

**Figure 7:**
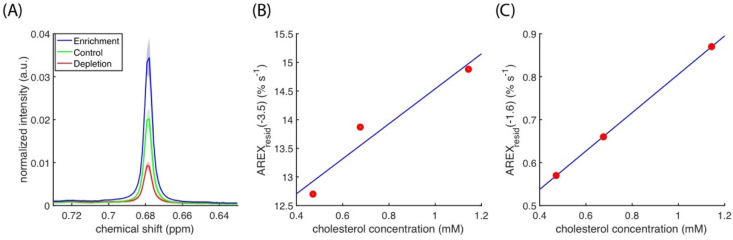
(**A**) Averaged ^1^H NMR signal (N = 3) of cholesterol methyl at 0.68 ppm from cholesterol-depleted (red), control (green), and cholesterol-enriched (blue) cell homogenates, in which standard deviations are indicated by shaded areas. Scattering plot illustrating the association between cholesterol concentration and AUCs of *AREX_resid_*(−3.5) (**B**) and *AREX_resid_*(−1.6) (**C**), in which regression is indicated by a blue line.

Figure 7A presents the spectra of cholesterol methyl at 0.68 ppm, and it indicates that cholesterol depletion and enrichment change the cholesterol concentration in cell cultures. The cholesterol concentration results indicated a linear correlation between the AUCs of *AREX_resid_*(−3.5) and *AREX_resid_*(−1.6) (Figure 7B,C). The linear regressions between cholesterol concentration and amides, amines, and rNOE at −1.6 and −3.5 ppm are listed in Table 1, and they suggest that amides, amines, and rNOE(−3.5) are slightly influenced by cholesterol concentration. The full ^1^H NMR spectra of the lipid extracts from the cell homogenates used in the CEST experiments are presented in Figure S5.

## 4. Discussion

Glutamate [3,14], creatine [1,4], and glucose [5,6]-weighted images can be generated using CEST techniques. These biomolecules are usually small and hydrophilic. Large hydrophilic biomolecules such as glycogen [2,48] can also be imaged using CEST techniques. Hydrophobic biomolecules are usually buried in biological membranes, in which molecular motions are hindered by surrounding lipids, resulting in a short molecular *T*_2_; furthermore, water accessibility can be highly reduced for molecules that are associated with membranes. These factors are deleterious to the formation of a CEST signal. The CEST signals that are related to the hydrophobic part of biomolecules are usually regarded as nonspecific (e.g., MT or rNOE at −3.5 ppm), and the studies of detecting hydrophobic molecules through CEST are still limited.

rNOE(−1.6) is specific to the choline in phosphatidylcholines; thus, it offers an opportunity to observe the changes that occur in biological membranes. A reduction of rNOE(−1.6) was detected in the brain tumor and stroke regions of the examined rats [9,24]. The liposome models indicated the presence of rNOE(−1.6) in cholesterol [25,26] and that the length and saturation degree of the hydrocarbon chains in phosphatidyl lipids were only slightly influenced by the magnitude of rNOE(−1.6) [25]. The hydroxyl in cholesterol was also revealed to be required for chemical exchanges [26]. However, the biological membranes comprised highly diverse lipids (including phosphatidylcholines) [49] and membrane proteins, and these components could influence the magnitude of rNOE(−1.6). The correlation between cholesterol content and rNOE(−1.6) in the biological models was not supported by direct evidence.

The amplitudes of rNOE(−1.6) in the z-spectra and *AREX_resid_* were reduced on the tumor side (Figure 1, Figure 2 and Figure 3); this finding is consistent with those reported by other studies [9,19,25]. Figure 4 presents the *AREX_resid_*(−1.6) images and the hematoxylin and filipin staining results together; they indicate that the tumor region was cholesterol-deficit and that the *AREX_resid_*(−1.6) in the tumor region was hypointense. We further demonstrated that the cholesterol concentration in the extracted tumors was lower than that in the normal tissues (Figure 5). A similar phenomenon was observed in the infrared images of the C6 tumors in rat brains in the literature [50], which had a higher cholesterol level in the peritumor region than in the tumor region. With respect to the cholesterol deficiency in the tumor region, our *AREX_resid_*(−1.6) images and filipin staining results are consistent with the infrared images produced in the literature [50]. Moreover, the average *AREX_resid_*(−1.6) (Figure 3B) and cholesterol quantification (Figure 5C) results did not reveal a significant longitudinal difference in the tumor region. These results indicated that glioma cells suppress their cholesterol synthesis pathway and uptake cholesterol through the LDL from nearby astrocytes [38]. 

We further verified the dependence of *AREX_resid_*(−1.6) on the cholesterol in C6 cell cultures (Figure 6), in which cholesterol levels can be reduced or increased by using MβCD or chol-MβCD solutions, respectively. The change in cholesterol level was verified through ^1^H NMR spectroscopy (Figure 7). The *AREX_resid_*(−1.6) of the cell homogenates has a positive correlation with cholesterol concentration, indicating that cholesterol enhanced the rNOE(−1.6) in the biological-derived samples; this finding is similar to that obtained from model liposomes [25,26]. This finding also supports the hypothesis that cholesterol increases the rNOE(−1.6) in animal models. 

The experiments of cell homogenate suggested that in addition to rNOE(−1.6), other apparent CEST signals (rNOE(−3.5), amide, and amine) had positive correlations with cholesterol content (Figure 6, Figure 7 and Figure S4; Table 1). The cavity enclosed by MβCD can accommodate a cholesterol molecule. Therefore, the magnitude of rNOE(−1.6) is presumably influenced by the cholesterol depletion and enrichment through MβCD and chol-MβCD, respectively. The cholesterol dependency of rNOE(−3.5) was reported by other studies, and it could be a result of the change in the order or fluidity of membranes [25,26]. Similarly, a low cholesterol dependency of amides and amines could be due to the membranes associated with biomolecules (e.g., membrane proteins). The amount of cholesterol (Figure 5) and the magnitudes of rNOE (Figure 3A,B) were also positively correlated with the animal models. Through our current protocol, we could visually separate the normal and tumor tissues when a tumor was sufficiently large. Therefore, we only obtained the lipid extracts on days nine and 12, which prevented us from performing an appropriate regression analysis of the association between cholesterol content and rNOE amplitude in the tissue studies. Also, in order to obtain the largest possible tumor tissues for the extraction, some of the surrounding normal tissues might not be fully removed. This contamination from the normal tissues is presumably increasing the cholesterol concentration of the tumor tissues. It might explain that the cholesterol concentration was roughly the same in the tumors between days nine and 12 (Figure 5C) instead of showing a decreasing trend (Figure 2K,L and Figure 3D).

Although we consistently found the cholesterol dependency of rNOE(−1.6), the ^1^H resonance suggests that the amounts of phosphatidylcholine were reduced in the tumors (Figure 5E), which was also found in the studies using infrared images [50]. Meanwhile, the amounts of total lipids remain roughly the same in tumor and normal tissues (Figure 5F). It suggests that both the cholesterol and phosphatidylcholine contents were reduced in the membranes of the tumor. The rNOE(−1.6) originates from the saturation of the choline methyls from the phosphatidylcholines, and hence the rNOE(−1.6) reduction in the glioma model might not be solely dependent on the cholesterol content. The reduction of rNOE(−1.6) was also observed in the ischemic stroke rat model [24], and the mechanism is unclear. We are not aware of a study showing the change in the cholesterol content during the period of ischemic stroke. However, it has been reported that the formation of free radicals could oxidize the lipid during ischemic stroke [51]. The change in the lipid compositions could be the possible source of reducing rNOE(−1.6).

Among the CEST effects, the magnitude of rNOE(−1.6) was small, and multiple-step processing was required to extract this information. In the present study, we used the *AREX_resid_* metric suggested by Zu [17] that the direct water saturation and MT is removed by a two-pool Lorentzian model, and the influence of water *T*_1_ is minimized by using the AREX method. Through the removal of rNOE(−3.5) from averaged residual rNOEs, the changes at approximately −1.6 ppm can be observed. However, our results were still considerably influenced by our choice of fitting conditions. For instance, the baseline of rNOE(−1.6) could be negative if the range of direct saturation was overestimated. With our fitting condition, the potential glycoNOE^2^ at −1 ppm was difficult to verify in *AREX_resid_*. Furthermore, the harvested cell cultures contained excess water, resulting in a considerable variation in wet weight, such that we had to quantify cholesterol by concentration per sample instead of cholesterol weight per unit of cell wet weight. Therefore, although we discovered a correlation between cholesterol content and rNOE(−1.6), we still lacked a quantified index that was appropriate for pathological evaluation. We attempted to integrate the results obtained from various experiments. Limits in experimental design meant that three groups of mice were required, specifically for CEST and MRI imaging (N = 9), tissue staining (N = 1), and tissue extraction (N = 3), all of which introduced additional variations. 

Monitoring the cholesterol homeostasis usually relies on the expression levels of the associating biomarker. For instance, the down-regulated LDL receptor (LDLR) and upregulated ABCA1 (ATP Binding Cassette Subfamily A Member 1) suggest that depleting cholesterol results in the vulnerable glioma cells [38]. Aside from cancer therapies [34,37,38], cholesterol metabolism is also essential to the neurodevelopment [52,53] and neurodegeneration [54,55]. The cholesterol-related activities can be monitored by the expression level of the associated proteins, including LRPs (low-density lipoprotein receptor-related proteins), uptaking LDL [52,53,54], or BMPs (bone morphogenetic proteins), signaling the cholesterol synthesis [55]. However, the techniques to evaluate the expression levels of cholesterol-associated biomarkers are usually only available for the cell cultures or ex vivo samples. Developing the in vivo imaging technique reflecting the cholesterol content is still needed. We hope that rNOE(−1.6) imaging could provide complementary information to the existing methods with the dependency on cholesterol.

## 5. Conclusions

Studies have suggested that interference with cholesterol homeostasis or metabolism is an effective therapy for brain cancer. However, an in vivo technique for imaging cholesterol has yet to be developed. We verified the positive correlation between cholesterol content and rNOE(−1.6) through multiple methods, including in vivo animal imaging, histology staining, use of cell homogenates, and tissue and homogenate extraction. However, the concentration of phosphatidylcholine also showed a positive correlation with rNOE(−1.6). Our studies suggest that the decreasing rNOE(−1.6) in the C6 glioma results from reduced cholesterol and phosphatidylcholine concentrations. Hence, it still requires more quantitative studies to reveal the meaning of the rNOE(−1.6) image at the molecular level.

## Figures and Tables

**Table 1:** Regression results for associations between cholesterol concentration and area under the curve (AUC) of the residuals of apparent exchange-dependent relaxation rate (*AREX_resid_*) in lipid extracts from cell homogenates.

	Slope (% s^−1^/mM)	Intercept (% s^−1^)	R^2^
rNOE(−3.5)	3.06	11.48	0.9310
rNOE(−1.6)	0.47	0.36	1.000
Amine	0.97	5.20	0.9838
Amide	0.45	2.03	0.9159

## Data Availability

The datasets generated during and/or analyzed during the current study are available from the corresponding author upon reasonable request.

## Supplementary Materials

The following supporting information can be downloaded at: https://www.mdpi.com/article/10.3390/biomedicines10061220/s1, Figure S1: Average z-spectra from normal and tumorous brain as measured on day 12 with various saturation amplitudes; Figure S2: T_2_-weighted, T_1_ Map, MT, and *AREX_resid_* (at 3.5, 2, −1.6, and −3.5 ppm) images of the mice shown in Figure 4; Figure S3: Averaged ^1^H NMR spectra from the normal and tumorous brain tissues; Figure S4: Average z-spectra from cholesterol-depleted, control, and cholesterol-enriched cell homogenates with various saturation amplitudes; Figure S5: Averaged ^1^H NMR spectra from cholesterol-depleted, control, and cholesterol-enriched cell homogenates.
