# Supplementary material for: Chemical Exchange Saturation Transfer (CEST) Signal at −1.6 ppm and Its Application for Imaging a C6 Glioma Model
Source: Biomedicines. 2022 May 24;10(6):1220. doi: 10.3390/biomedicines10061220 (PMC9219881; doi:10.3390/biomedicines10061220)
Supplement: Supplementary file 1 [file biomedicines-10-01220-s001.zip › biomedicines-1706828-supplementary.pdf]

Supporting information

Chemical Exchange Saturation Transfer (CEST) Signal at −1.6 ppm and Its Application for Imaging a C6 Glioma Model

Qi-Xuan Wu[1, 2], Hong-Qing Liu[1, 2], Yi-Jiun Wang[1], Tsai-Chen Chen[2], Ziying Wei[1, 2], Jung-Hsuan Chang[1], Ting-Hao Chen[1], Jaya Seema[2], and Eugene C. Lin*[1, 3]

[1]Department of Chemistry and Biochemistry, National Chung Cheng University, Chiayi, Taiwan,

[2]Institute of Biomedical Sciences, Academia Sinica, Taipei, Taiwan

[3]Center for Nano Bio-detection, National Chung Cheng University, Chiayi, Taiwan

Detailed fitting procedure

MRI

(1) Fitting water and MT in a z-spectrum

The offsets concerning water and MT (±9, ±7, and −0.5~0.5 ppm with a 0.1-ppm increment) from a z-spectrum were fitted based on two Lorentzian functions,

$$S(\Delta\omega) = 1 - f_w \frac{(0.5W_w)^2}{(\Delta\omega - c_1)^2 + (0.5W_w)^2} - f_m \frac{(0.5W_m)^2}{(\Delta\omega - c_2)^2 + (0.5W_m)^2} \quad \text{(S1)}$$

f, W, and c are the amplitude, width, and center of a saturation dip, respectively, the subscribed w and m are water and MT pools, respectively, and Δω is the saturation offset. The MT pool size in Equation 2 was obtained from the fitted f_m in Equation S1. The initial and boundary conditions are listed in Table S1.

Table S1 The initial condition for fitting water and MT from a z-spectrum

	f_w	W_w (ppm)	c_w (ppm)	f_m	W_m (ppm)	c_m (ppm)
Initial condition	0.9	1	0	0.5	10	−2
Lower boundary	0	0.1	−0.3	0	5	−4
Upper boundary	1	3	0.3	1	40	4

(2) $AREX_{resid}$(−3.5) isolation

The $AREX_{resid}$(−3.5) was isolated from a range between −3 to −4 ppm of the $AREX_{resid}$ spectrum by a Gaussian function,

$$AREX_{resid}(\Delta\omega) = a \exp(-(\frac{\Delta\omega - b}{c})^2). \quad \text{(S2)}$$

a is the amplitude, b is the center, and c is the width. The fitting range was between −3 to −4 ppm. The fitting conditions are listed in Table S2.

Table S2 The initial condition to isolate $AREX_{resid}$(−3.5)

	a (s^{-1})	b (ppm)	c (ppm)
Initial condition	$AREX_{resid}$(−3.5)	−3.5	1
Lower boundary	0.5×$AREX_{resid}$(−3.5)	−3.8	0.5
Upper boundary	2×$AREX_{resid}$(−3.5)	−3.2	2

NMR

(1) T_1 fitting in cell homogenates

Equations S3 and S4 are identical to Equations 3 and 4, respectively, which were utilized to address T_1 relaxation from two pools.

$$I(t) = 1 - 2(x_a \exp\left(-\frac{t}{T_{1,a}}\right) + (1 - x_a)\exp\left(-\frac{t}{T_{1,b}}\right)) \quad \text{(S3)}$$

$$T_{1,avg} = x_a T_{1,a} + (1 - x_a)T_{1,b} \quad \text{(S4)}$$

I(t) is the water intensity with delay t, $T_{1,a}$ and $T_{1,b}$ is the T_{1s} for pool a and b, respectively, x_a is the portion of a, and $T_{1,avg}$ is the averaged T_1. The fitting results are listed in Table S3.

Table S3. The fitting results and the averaged T_1 of the cell homogenates.

	Sample	$T_{1,a}$ (ms)	$T_{1,b}$ (ms)	x_a (%)	R^2	$T_{1,avg}$ (ms)
Cholesterol depletion	1	2978	195	36.8	0.985	1220
	2	2970	75	28.4	0.991	898
	3	3006	162	32.4	0.989	1084
Cholesterol enrichment	1	2970	174	37.1	0.990	1210
	2	2949	172	36.5	0.990	1186
	3	3256	128	37.7	0.991	1203
Control	1	3123	133	28.3	0.990	978
	2	3051	141	34.5	0.993	1145
	3	3186	111	35.5	0.991	1305

(2) AREX correction in cell homogenates

The offsets concerning water and MT (±20, ±15, ±10, ±6, and −0.8~0.8 ppm with a 0.1-ppm increment) from a z-spectrum were fitted based on Equation S1. The initial conditions utilized in the fitting are listed in Table S4.

Table S4 The initial condition for fitting water and MT from a z-spectrum

	f_w	W_w (ppm)	c_w (ppm)	f_m	W_m (ppm)	c_m (ppm)
Initial condition	0.95	0.5	0	0.05	15	0
Lower boundary	0.9	0.1	−1	0	2	−4
Upper boundary	1	2	1	0.2	50	4

(3) rNOE isolations in cell homogenates

The $AREX_{resid}(-3.5)$ was isolated from a range between −0.8 to −4.5 ppm of the $AREX_{resid}$ spectrum by two Gaussian functions,

$$AREX_{resid}(\Delta\omega) = a_1 \exp(-(\frac{\Delta\omega - b_1}{c_1})^2) + a_2 \exp(-(\frac{\Delta\omega - b_2}{c_2})^2). \quad \text{(S5)}$$

a is the amplitude, b is the center, and c is the width, and subscripts 1 and 2 indicate the rNOE at −3.5 and −1.6 ppm, respectively. The fitting conditions are listed in Table S5.

Table S5 The initial condition to isolate $AREX_{resid}(-3.5)$ and $AREX_{resid}(-1.6)$

<table>
<tr><th></th><th>a_1 (s^{-1})</th><th>b_1 (ppm)</th><th>c_1 (ppm)</th><th>a_2 (s^{-1})</th><th>b_2 (ppm)</th><th>c_2 (ppm)</th></tr>
<tr><td>Initial condition</td><td>$AREX_{resid}(-3.5)$</td><td>−3.5</td><td>1</td><td>$AREX_{resid}(-1.6)$</td><td rowspan="3">Fixed at −1.6</td><td>1</td></tr>
<tr><td>Lower boundary</td><td>$0.5 \times AREX_{resid}(-3.5)$</td><td>−3.8</td><td>0.1</td><td>$0.5 \times AREX_{resid}(-1.6)$</td><td>0.1</td></tr>
<tr><td>Upper boundary</td><td>$1.5 \times AREX_{resid}(-3.5)$</td><td>−3.2</td><td>2</td><td>$1.5 \times AREX_{resid}(-1.6)$</td><td>2</td></tr>
</table>

Figure

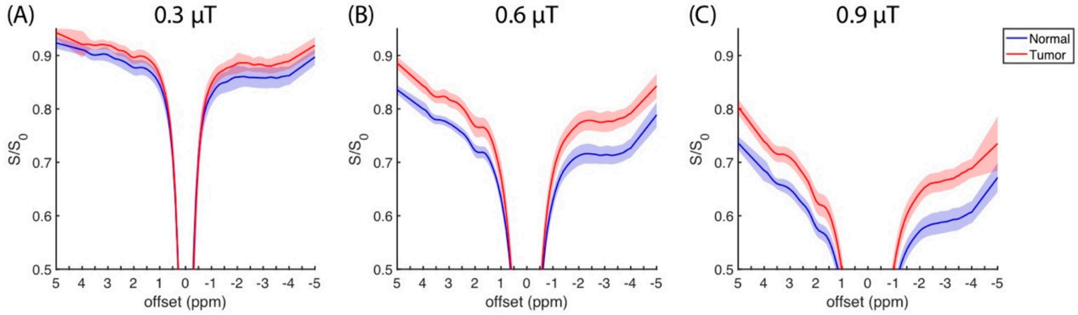

Figure S1. Average z-spectra (N = 9) from the normal (blue) and tumor (red) brain measured on day 12. Shading areas indicate standard deviation resulting from animal variation. The saturation amplitude is 0.3 μT (A), 0.6 μT (B), and 0.9 μT (C), and the saturation duration is 4 s.

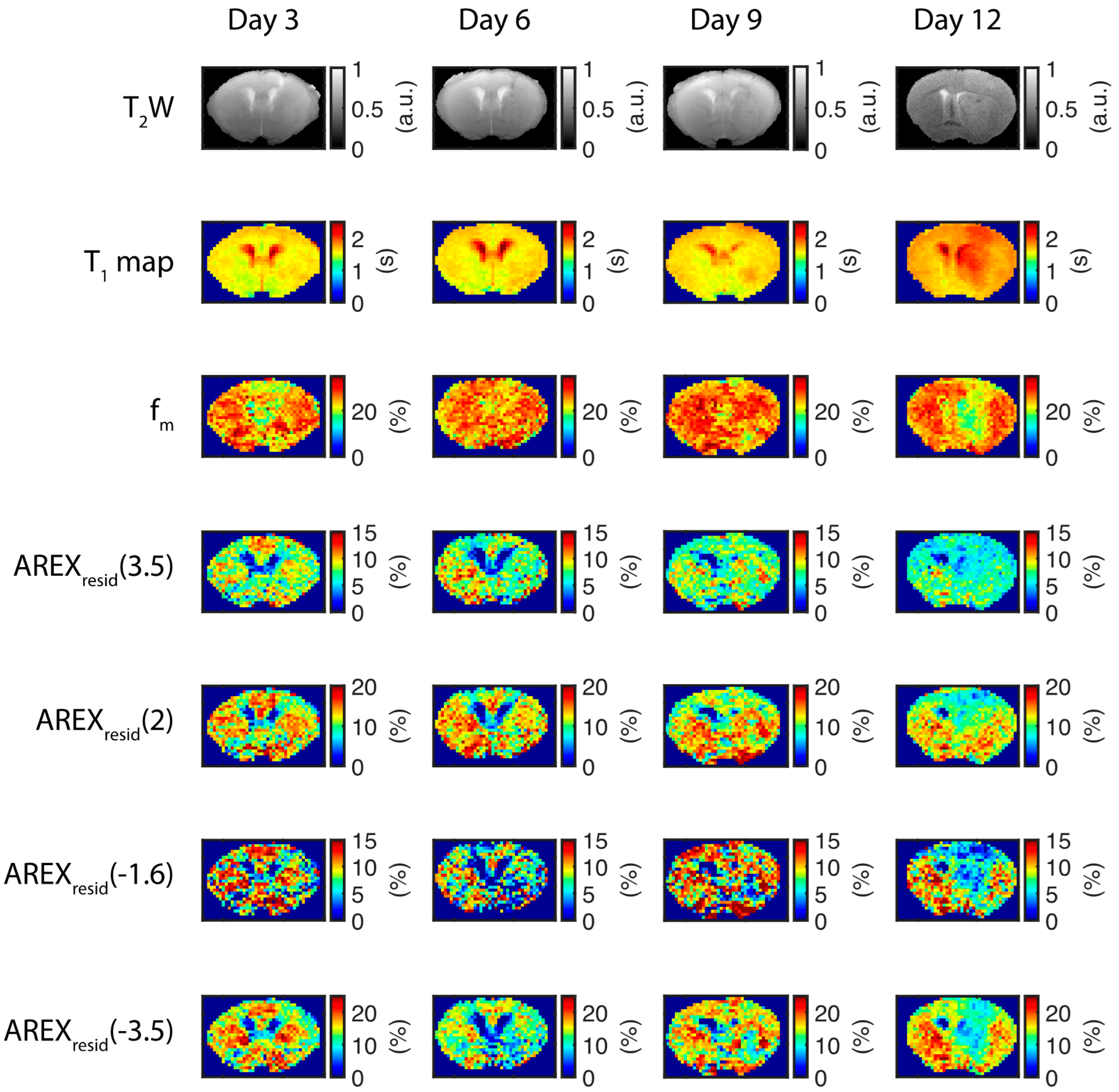

Figure S2 The T_2-weighted, T_1 Map, MT, and $AREX_{resid}$ (at 3.5, 2, −1.6, and−3.5 ppm) images of the mice shown in Figure 4.

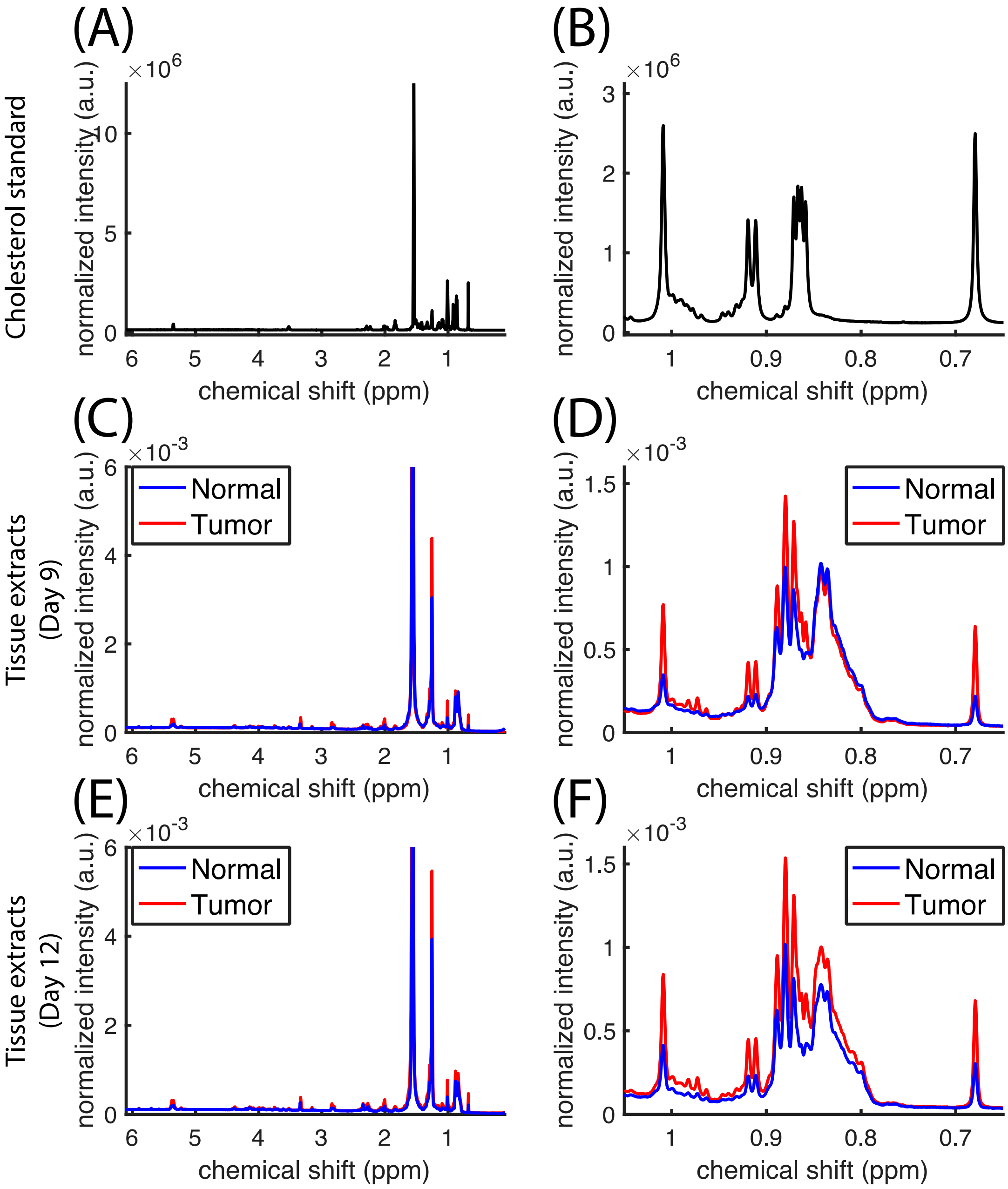

Figure S3. The ^{1}H NMR spectra of the cholesterol standard (A) and (B), and the averaged ^{1}H NMR spectra (N = 3) of lipid extracts from the normal and tumor brain tissues (C)-(F). The full range spectra are shown in (A), (C), and (E), and the enlarged spectra are shown in (B), (D), and (F), indicating the region with the cholesterol characteristic peaks.

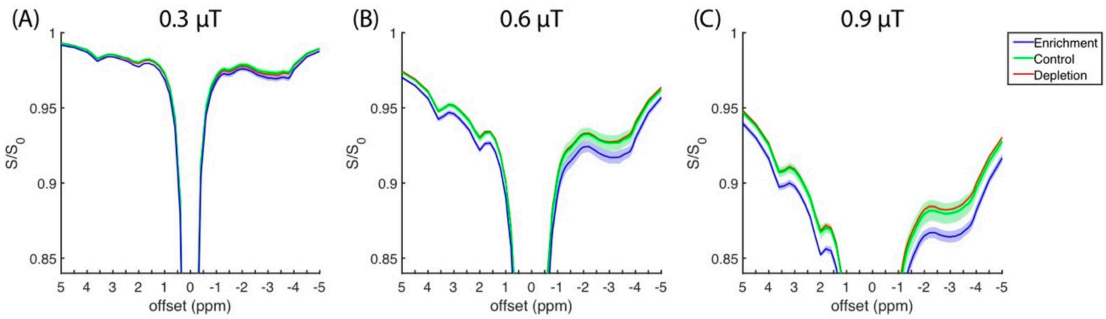

Figure S4. The averaged z-spectra (N = 3) from the cholesterol-depleted (red), control (green), and cholesterol-enriched (blue) cell homogenates with a saturation amplitude of 0.3 µT (A), 0.6 µT (B), and 0.9 µT (C), and the saturation duration is 5 s.

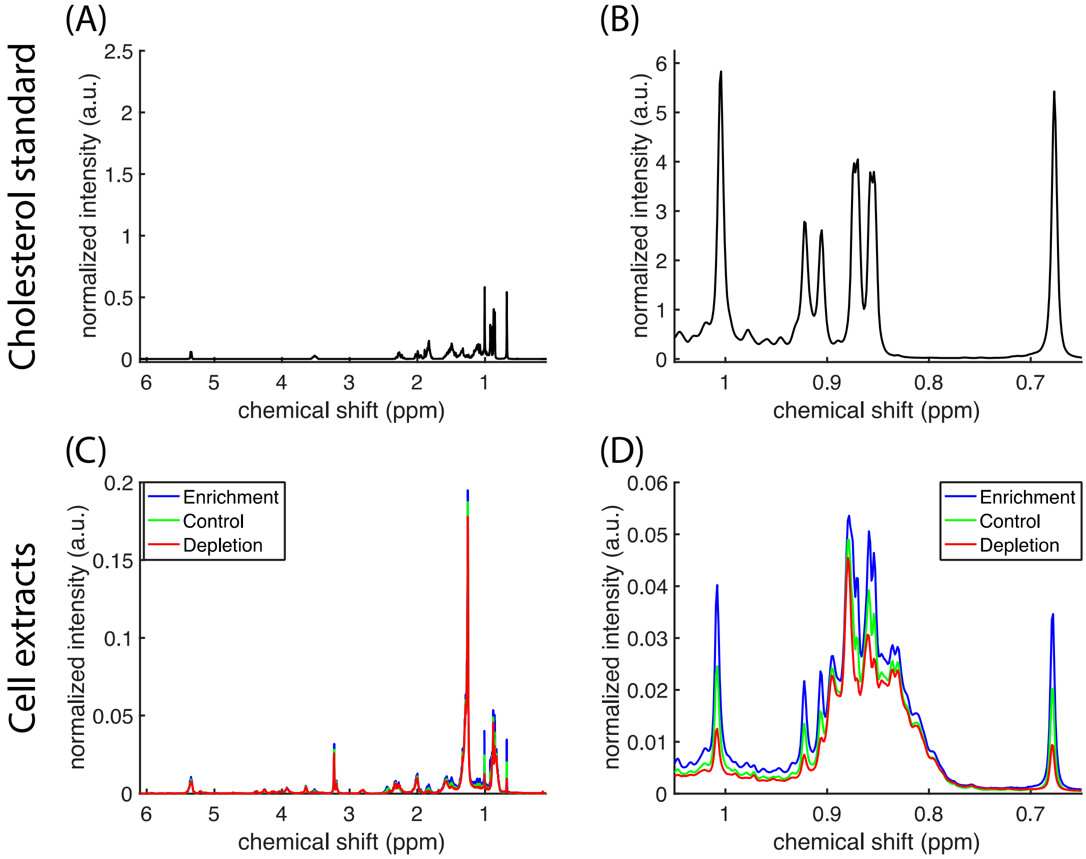

Figure S5. The averaged ^{1}H NMR spectra (N = 3) of lipid extracts from the chol-depleted (red), control (green), and chol-enriched (blue) cell homogenates (C) and (D), and the ^{1}H NMR spectra of the cholesterol standard (A) and (B). The full range spectra are shown in (A) and (C), and the enlarged spectra are shown in (B) and (D), indicating the region with the cholesterol characteristic peaks.
